# Highly reduced-dose CT of the lumbar spine in a human cadaver model

**DOI:** 10.1371/journal.pone.0240199

**Published:** 2020-10-08

**Authors:** Malte Lennart Warncke, Nis Jesper Wiese, Enver Tahir, Susanne Sehner, Axel Heinemann, Marc Regier, Klaus Püschel, Gerhard Adam, Julius Matthias Weinrich, Azien Laqmani

**Affiliations:** 1 Department of Diagnostic and Interventional Radiology and Nuclear Medicine, University Medical Centre Hamburg-Eppendorf, Hamburg, Germany; 2 Department of Medical Biometry and Epidemiology, University Medical Centre Hamburg-Eppendorf, Hamburg, Germany; 3 Department of Legal Medicine, University Medical Centre Hamburg-Eppendorf, Hamburg, Germany; Humanitas Clinical and Research Center—IRRCS, ITALY

## Abstract

**Purpose:**

Feasibility of a highly reduced-dose lumbar spine CT protocol using iterative reconstruction (IR) in a human cadaver model.

**Materials and methods:**

The lumbar spine of 20 human cadavers was repeatedly examined using three different reduced-dose protocols (RDCT) with decreasing reference tube current-exposure time products (RDCT-1: 50 mAs; RDCT-2: 30 mAs; RDCT-3: 10 mAs) at a constant tube voltage of 140 kV. A clinical standard-dose protocol (SDCT) served as the reference (reference tube current–exposure time product: 70 mAs; tube voltage: 140 kV). Images were reconstructed using filtered back projection (FBP) and two increasing levels of IR: IRL4 and IRL6. A five-point scale was used by two observers to assess the diagnostic quality of anatomical structures (cortical and trabecular bone, intervertebral foramina, pedicles and intervertebral joints, spinous and transverse processes). Objective image noise (OIN) was measured. Results were interpreted using a linear mixed-effects regression model.

**Results:**

RDCT-2 with IRL6 (1.2 ± 0.5mSv) was the lowest reduced-dose protocol which provided diagnostically acceptable and equivalent image quality compared to the SDCT (2.3 ± 1.1mSV) with FBP (p > 0.05). All RDCT protocols achieved a significant reduction of the mean (±SD) effective radiation doses (RDCT-1: 1.7±0.9mSv; RDCT-2: 1.2±0.5mSv; RDCT-3: 0.4±0.2mSv; p < 0.05) compared to SDCT. OIN was lower in all RDCT protocols with the application of IRL4 and IRL6, compared to the SDCT with FBP (p < 0.05).

**Conclusion:**

Highly reduced-dose lumbar spine CT providing diagnostically acceptable image quality is feasible using IR in this cadaver model and may be transferred into a clinical setting.

## Introduction

Plain radiography of the skeleton, including the lumbar spine, is commonly performed, accounting for nearly 30% of all radiographic exams ordered in Germany [1–3]. In comparison with CT and MRI, the limitations of this imaging modality are well known [4–6]. For example, with CT the sensitivity (100%) and specificity (97%) in the detection of thoracolumbar fractures is excellent, whereas plain radiographs are reported to be only 73% sensitive and 100% specific [7]. The radiation dose of a two-view radiograph of the lumbar spine is reported to be around 1.1 mSv [1]. However, lumbar spine CT exposes patients to high doses of ionizing radiation, with reported values up to 19 mSv [8,9]. It has been postulated that increasing radiation exposure leads to a higher risk of inducing neoplasms [10,11]. Following the ALARA-principles (as low as reasonably achievable), a reduction of ionizing radiation in standard dose CT (SDCT), without compromising image quality, is required [12]. Dose reduction alone is associated with increasing image noise, resulting in impaired image quality.

Iterative reconstruction (IR) algorithms are commonly used to compensate for the higher image noise and the frequently associated impaired image quality in reduced-dose CT (RDCT). IR may allow further dose reduction, resulting in dose exposure levels that are comparable to that of plain radiographs [13–16]. Since there is a steady increase in performance of CT examinations, the implementation of reduced-dose CT scanning in clinical practice is highly desirable [1,17].

Apart from two prospective studies (one demonstrating the higher diagnostic value of low dose lumbar spine CT versus plain radiographs; the other showing that diagnostically acceptable lumbar spine CT in human beings is feasible at an effective dose of 2.6 ± 0.9 mSv), solely retrospective studies have been published [18–22].

To the best of our knowledge, a prospective study of a stepwise dose reduction in lumbar spine CT protocols using IR has not been performed in a human cadaver model.

The aim of this study was to investigate the feasibility of a diagnostically acceptable highly reduced-dose lumbar spine CT protocol in a human cadaver model that results in radiation exposure levels that are comparable to that of plain radiographs.

## Materials and methods

The local ethics committee (Ärztekammer Hamburg, Germany) approved the study (WF33/17). All data was utilized anonymously.

### Study population

20 human cadavers (11 male and 9 female) from the department of legal medicine of our institution were scanned between February and June 2017. Inclusion criteria comprised informed consent from the relatives of the deceased individual and age older than 18 years. Exclusion criteria were known bone diseases (eg metabolic bone disease or bone metastases), severely traumatic injuries, and/or any high density foreign bodies in the scan volume (e.g. metallic implants, stent material, vascular implants, bone cement) [23,24]. The average BMI of the 20 human cadavers was 26.3 ± 4.5 kg/m^2^ (average height: 171.1 ± 9.1 cm; average weight: 77.6 ± 17.8 kg). The average age of death was 70.0 ± 15.9 years. CT scans were performed at a mean of 4.6 ± 4.9 days post mortem. None of the cadavers had underlying metabolic bone disease or osseous neoplasia.

### Imaging protocols

All scans were performed on a 256-slice CT scanner (Brilliance iCT, Philips Healthcare) using the following parameters: tube voltage: 140 kV; detector collimation: 64 x 0.625 mm; pitch: 0.579; tube rotation time: 0.5 s. The SDCT employed a reference tube current-exposure time product of 70 mAs. Three decreasing RDCT protocols with the following reference tube current-exposure time products were performed: RDCT-1: 50 mAs, RDCT-2: 30 mAs and RDCT-3: 10 mAs (Table 1). The automatic exposure control system (automatic current selection) combined with z-axis dose-modulation (Z-DOM) was used.

**Table 1:** CT protocol parameters.

	SDCT	RDCT-1	RDCT-2	RDCT-3
Reference tube current-exposure time product (mAs)	70	50	30	10
Tube voltage (kV)	140			
Collimation (mm)	64 x 0.625			
Rotation time (s)	0.5			
Pitch	0.579			
Automatic tube current modulation	Automatic current selection with z-axis dose modulation (Z-DOM, Philips Healthcare)			
Level of iteration	FBP and IR levels 4 and 6			
Kernel	Bone (D)			
Section thickness (mm)	3			
Reformations	Axial, coronal, sagittal			

SDCT = standard-dose CT; RDCT = reduced-dose CT; FBP = filtered back projection; IR = iterative reconstruction.

The specimens were examined in supine position with the head first on the CT table. The arms were placed overhead for all scans. The scan range was defined based on a lateral topogram.

### Reconstruction techniques

All CT datasets were reconstructed using FBP and two increasing levels (IRL4 and IRL6) of an established IR algorithm (iDose^4TM^, Philips Healthcare) [25]. iDose^4TM^ corrects CT measurements with a poor signal-to-noise ratio or low photon counts within the projection dataset. The remaining localized noise is then reduced by a structure model fitting within the image space.

Increasing levels of iDose^4TM^ produce higher levels of noise reduction. The algorithm is already in clinical use and allows for image reconstruction within under a minute. Further technical details of IR have been described elsewhere [26].

**Fig 1:**
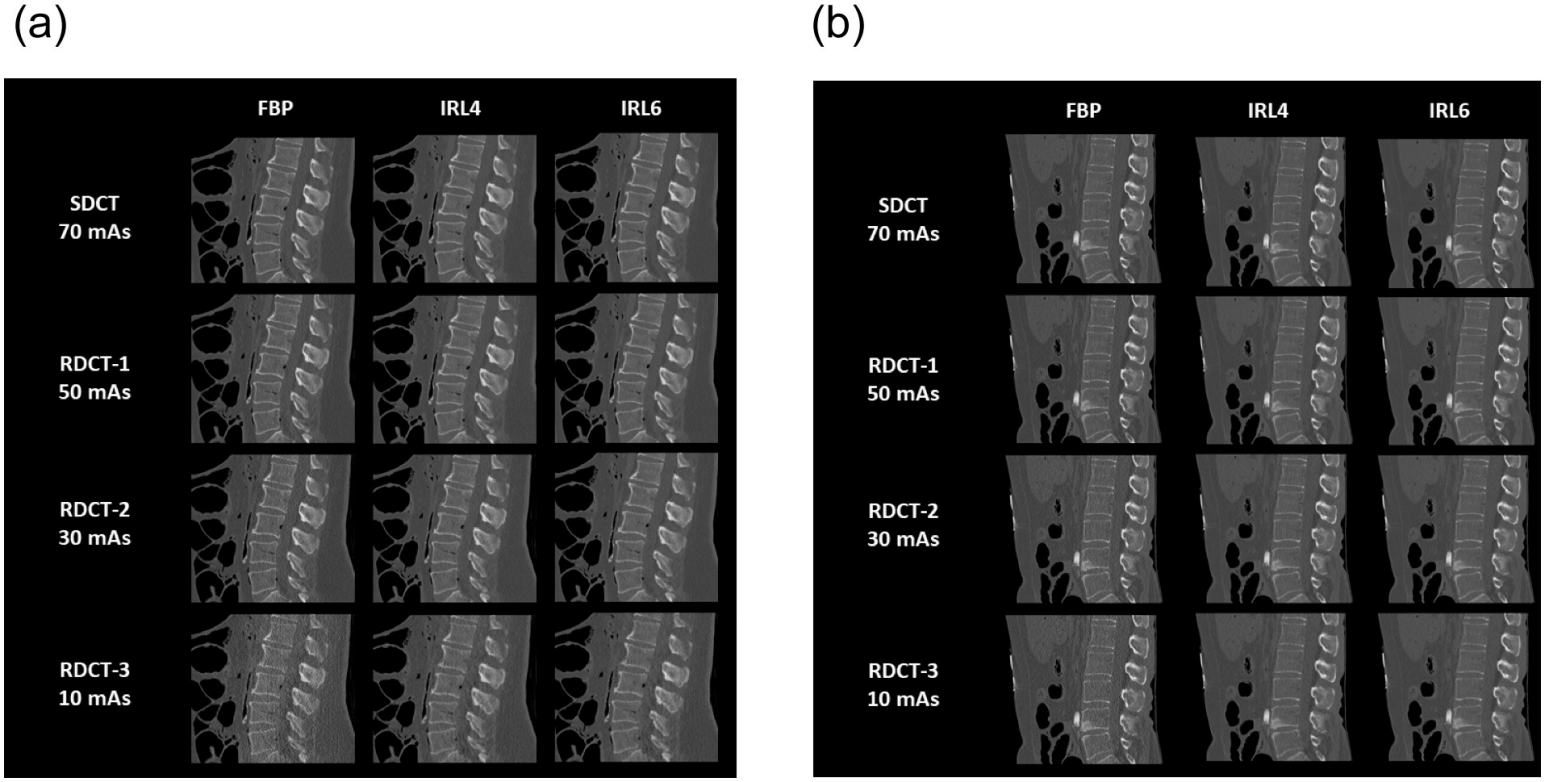
a. All CT protocols (SDCT and RDCT-1, 2 and 3) reconstructed with FBP and IRL4 and IRL6 in sagittal reformations. b. As for Fig 1a, but images were obtained from a male cadaver (age at death: 53 years; BMI: 20.8 kg/m^2^).

All datasets were reconstructed using a bone kernel with a slice thickness of 3 mm. Finally, images were reconstructed in the axial, coronal and sagittal planes (Fig 1a and 1b).

Images were obtained from a female cadaver (age at death: 54 years; BMI: 29.4 kg/m^2^). Subjective image quality ratings were high for SDCT, RDCT-1 and RDCT-2 with IRL4 and IRL6, leading to a diagnostically acceptable image quality in all protocols. RDCT-2 with FBP and all RDCT-3 protocols were rated as unacceptable for diagnostic purposes in this instance.

In this case, the protocols SDCT, RDCT-1, RDCT-2 and RDCT-3 with IRL6 were rated as diagnostically acceptable. RDCT-3 with FBP and IRL4 produced insufficient diagnostic image quality.

### Radiation dose

The CT dose index (CTDI_vol_) and dose-length product (DLP) were obtained from the automatically generated dose protocol of each examination. The effective radiation dose (ED) was calculated by multiplying the DLP (mGy × cm) by a conversion factor of 0.015 mSv/(mGy × cm) [27].

Furthermore, size-specific dose estimates (SSDE) were calculated for all subjects. As a prerequisite, the effective diameter from the anteroposterior (AP) and lateral (LAT) dimensions at the third lumbar vertebra was determined:

$$\text{Effective}\;\text{diameter}\;(\text{cm})\;=\;\sqrt{\text{AP}}\;\times \;\text{LAT}$$

Each conversion factor was based on the Medicine Report No. 204 by the American Association of Physicists [28]. SSDE was calculated as follows:

$$\text{SSDE}\;(\text{mGy})\;={\;\text{CTDI}}_{\text{vol}}\;\times \;\text{conversion}\;\text{factor}$$

### Quantitative image analysis

Objective image noise (OIN) measurements were performed on a dedicated PACS workstation (PACS IW, GE Healthcare).

**Fig 2:**
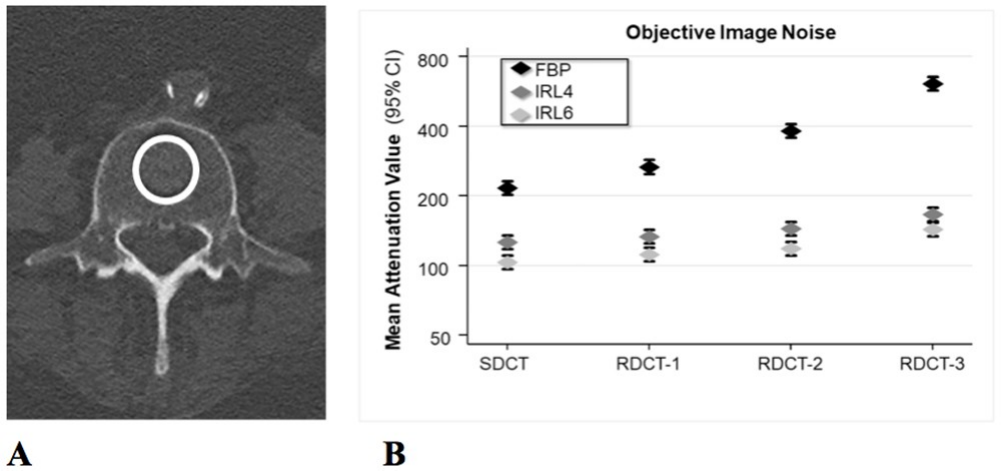
Objective image noise. **A** Measurement of CT number and objective image noise (OIN). Axial CT image shows placement of ROI (circle) in the center of the body of the third lumbar vertebra. Measurements were performed in three adjacent slices and the mean values were calculated. **B** Quantitative analyses of OIN (defined as SD of CT number) for SDCT and three different RDCT protocols, as measured in the center of the body of the third lumbar vertebra. Images were reconstructed with FBP and IRL4 and IRL6. Horizontal midpoints of diamonds represent mean values, and error bars denote 95% CIs. Compared to SDCT with FBP, even the RDCT protocol with lowest dose (RDCT-3) reconstructed with IR resulted in lower OIN.

CT numbers (CT-N), defined as the attenuation in Hounsfield units (HU), were measured in the axial plane by placing circular regions of interest (ROIs) of 30 mm^2^ in the center of the third lumbar vertebra in each subject for each protocol (Fig 2). In order to prevent potential bias in a single measurement, each region was measured at three adjacent slices [23]. Results were averaged for further analyses. The standard deviation (SD) of CT-N served as the OIN [20].

### Qualitative image analysis

All CT datasets were anonymized, randomized and reviewed in a blinded manner. The images were reviewed independently by two observers with 4 and 5 years of experience respectively in musculoskeletal imaging. Images were reviewed using a bone window setting (window width: 2500 HU; window center: 500 HU). Each observer was allowed to review all reconstructions (axial, sagittal and coronal) for each paired image set. The image quality of defined anatomical structures (cortical and trabecular bone, intervertebral foramina, pedicles and intervertebral joints, spinous and transverse processes) was separately rated using a modified five-point scale [15]:

1. poor image quality, major artefacts, major image noise
2. reduced image quality, substantial artefacts, extensive image noise
3. acceptable image quality, moderate artefacts, moderate image noise
4. good image quality, minor artefacts, minor image noise
5. excellent image quality, no artefacts, no perceived image noise

Scores of 1 and 2 were defined as not acceptable for diagnostic purposes, whereas scores of 3 to 5 were considered as diagnostically acceptable.

### Statistical analysis

Sample characteristics are given as absolute and relative frequencies, or mean +/- standard deviation, whichever is appropriate. A mixed effect model for repeated measures (MMRM) was used to account for the repeated measurement structure of the data.

The effect of reconstruction level on the quantitative parameters (CT-N and OIN) was evaluated accordingly. Three adjacent image slices per subject and reconstruction level were defined. Based on this definition, a random intercept for each subject was modelled.

The overall image quality of the anatomical structures was modelled concurrently to the quantitative parameters. A second random intercept term for the readers was included to account for potential cluster effects.

In order to compare the reconstruction levels within a protocol for quantitative and qualitative parameters, all factors and their interactions (including all consecutive interactions) were considered accordingly in the model. In the case of an insignificant interaction term, only the consecutive interactions or the main effects remained in the model. This decision was reached by using the likelihood ratio test for model comparison. Due to their ability to potentially affect the relationship between the three predictors and the outcome, the following variables were considered in the models: BMI, age, sex, days post mortem and scan length. An indicator for the anatomical structure was also added in this model, since the visibility was rated at three different anatomical structures. For all estimated models the sufficient model assumption of normal distributed residuals was checked. Results were reported as estimated marginal means, which are represented in graphs with their corresponding 95% confidence intervals (95% CIs). Because of the multiplicity of statistical tests resulting from diverse comparisons, Bonferroni test–adjusted p values and CIs were reported.

Interobserver agreement was assessed by intraclass correlation coefficient (ICC) analyses. p values < 0.05 were considered significant. All analyses were computed using Stata 15.1 (StataCorp).

## Results

### Radiation dose

The mean exposure, CTDI_vol_, DLP, SSDE and effective radiation dose were significantly lower in all three RDCT protocols (all p < 0.001) in comparison to SDCT (Table 2).

**Table 2:** Radiation dose.

Measurement	SDCT	RDCT-1	RDCT-2	RDCT-3
Tube current-exposure time product (mAs)^a^	65.6 *±* 33.0	46.5 *±* 23.9	27.0 *±* 14.3	12.5 *±* 9.4
CTDI_vol_ (mGy)	6.7 *±* 3.7	4.7 *±* 2.4	2.8*±* 1.5	1.1 *±* 0.4
DLP (mGy*cm)	148.3 *±* 71.3	111.5 *±* 58.4	64.9 *±* 34.8	26.1 *±* 8.9
Effective dose (mSv)	2.3 *±* 1.1	1.7 *±* 0.9	1.2 *±* 0.5	0.4 *±* 0.2
SSDE (mGy)	7.9 *±* 2.6	5.5 *±* 2.2	3.5 *±* 1.1	1.4 *±* 0.6

Each table cell data is the mean (± SD). CTDIvol = volume CT dose index; DLP = dose-length product; SSDE = size-specific dose estimate.

^a^ Values are stated as modulated tube current values.

The effective radiation dose was reduced by 26% in RDCT-1, by 48% in RDCT-2 and by 83% in RDCT-3 when compared with SDCT.

### Quantitative results

The mean CT-numbers in SDCT and RDCT exams did not differ between protocols or reconstruction algorithms (p > 0.05).

BMI, age, sex, days post mortem and scan length did not significantly influence OIN.

OIN increased linearly with subsequent dose reduction steps for all reconstructions. Application of IR significantly decreased the OIN in comparison to FBP. With application of IR, OIN was lower in all RDCT protocols compared to the SDCT with FBP (p < 0.05) (Fig 2).

### Subjective image quality

**Fig 3:**
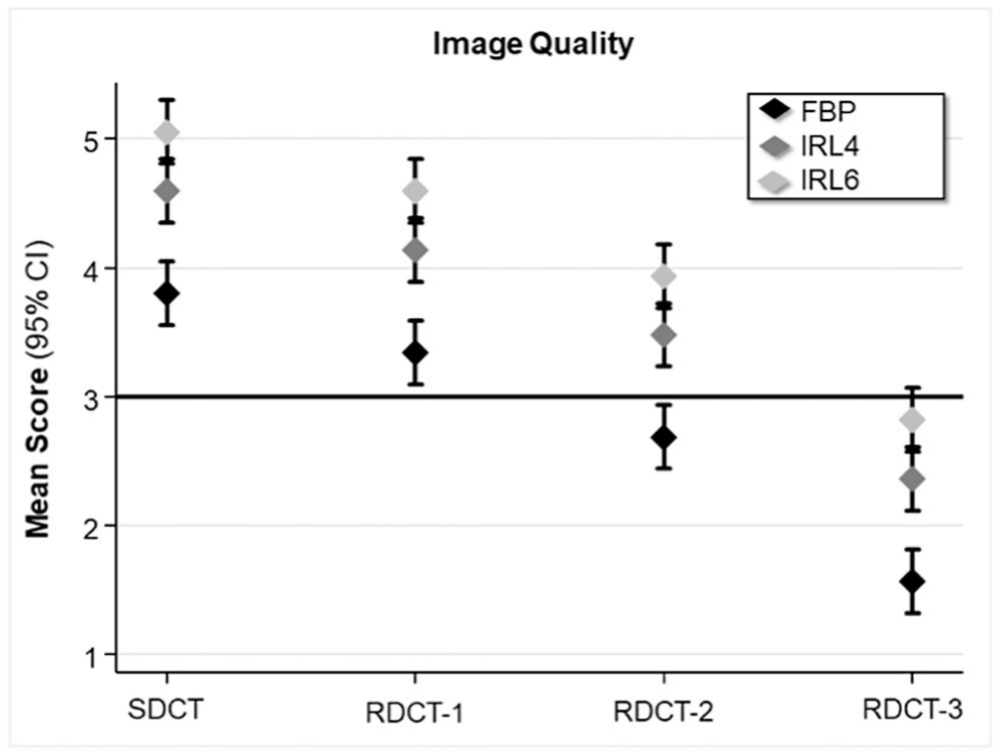
Mean image quality ratings of all analyzed anatomical structures for SDCT and RDCT protocols.

There was no difference in image quality ratings of the evaluated separate anatomical structures (p > 0.05). Ratings are presented as an average (Fig 3).

Images were reconstructed using FBP and IRL4 and IRL6. Error bars represent the 95% CI. The Y-axis depicts the subjective five-point grading scale (1 indicating worst through to 5 indicating best). Scores of 3 to 5 were considered as diagnostically acceptable image quality. The CT protocol with the lowest radiation dose allowing diagnostically acceptable image quality for all anatomical structures in all studies was RDCT-2 with IRL6.

Compared to FBP reconstructions, the image quality of IR was rated significantly higher for SDCT and all RDCT protocols (p < 0.05). There was no significant difference between the two applied strengths of IR (p > 0.05).

RDCT-2 with IRL6 was the lowest reduced-dose protocol which provided equivalent image quality to the SDCT with FBP (Fig 3).

### Diagnostic quality of anatomical structures

With application of IR, the image quality of all anatomical structures was rated as diagnostically acceptable for all cadavers for SDCT and RDCT-1 (Table 3).

**Table 3:** Number of diagnostically acceptable studies of the lumbar spine.

		SDCT			RDCT-1			RDCT-2^a^			RDCT-3		
		FBP	IRL4	IRL6	FBP	IRL4	IRL6	FBP	IRL4	IRL6	FBP	IRL4	IRL6
**Diagnostically Acceptable**	**Yes**	20	20	20	19	20	20	13	19	20	8	13	18
	**No**	0	0	0	1^b^	0	0	7^b^	1^b^	0	12^b^	7^b^	2^b^

Table cell data is number of individual studies.

FBP = filtered back projection; IR = iterative reconstruction.

^a^ RDCT-2 with IRL6 was the lowest reduced-dose CT protocol which produced diagnostically acceptable image quality for all anatomical structures in all studies.

^b^ Protocols and reconstructions whose image quality was not diagnostically acceptable for at least one anatomical structure (cortical and trabecular bone, intervertebral foramina, pedicles and intervertebral joints, spinous and transverse processes).

RDCT-2 with IR4 was not rated as diagnostically acceptable in one cadaver. When using FBP, only the SDCT led to a diagnostically acceptable image quality in all specimens.

**Fig 4:**
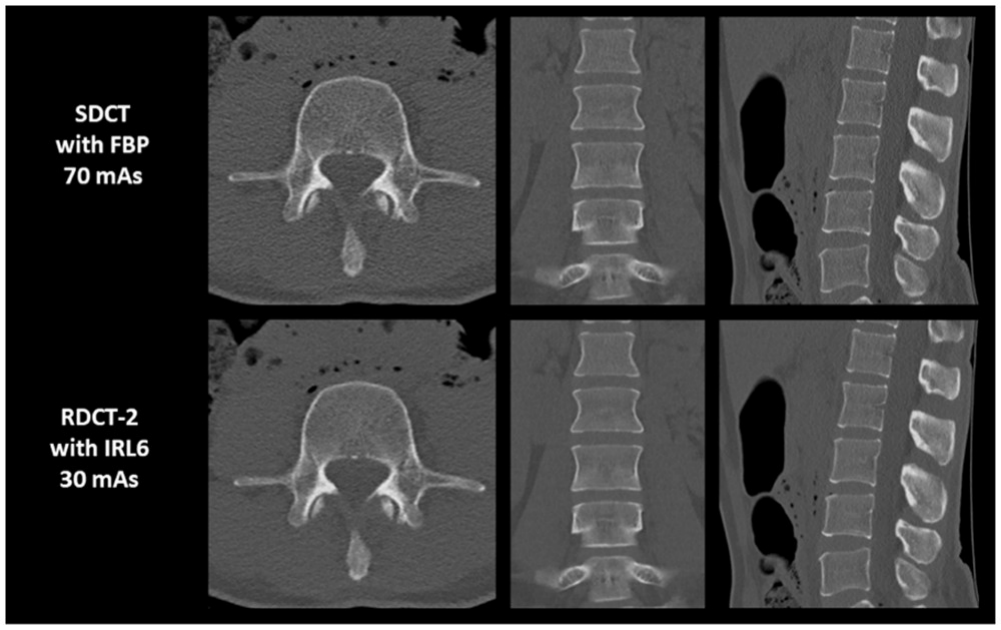
Direct comparison in quality of axial, coronal and sagittal reformatted images produced by the SDCT reconstructed with FBP, and RDCT-2 reconstructed with IRL6.

The CT protocol with the lowest radiation dose which provided diagnostically acceptable image quality of all anatomical structures in all specimens was RDCT-2 with IRL6 (Fig 3 and Table 3). A direct comparison of the SDCT reconstructed with FBP, and RDCT-2 reconstructed with IRL6, is displayed in Fig 4.

Images were obtained from a 65-year-old female cadaver with a BMI of 24.8 kg/m^2^. For both protocols, all anatomical structures were rated as diagnostically acceptable in all studies.

### Interobserver agreement

The two readers showed a high level of agreement in image quality of anatomical structures (ICC = 0.89; 95%-CI: 0.88–0.90).

## Discussion

Our results demonstrate that diagnostically acceptable highly reduced-dose CT of the lumbar spine in cadavers is feasible at 1.2 mSv using IR. The calculated effective radiation dose of our dose-reduced CT protocol (1.2 mSv) is similar to reported dose values of a two-view radiograph (1.1 mSv), while offering a substantially higher diagnostic value [1,4]. This claim is supported by a study published in 2016 that addressed dose exposure and image quality of plain radiographs vs RDCT, demonstrating the diagnostic superiority of CT [21]. Hence, plain radiographs of the lumbar spine may be replaced by our reduced-dose CT protocol in clinical practice.

Our proposed imaging protocol reduces the effective radiation dose by 48% in comparison with our clinically established SDCT. European and American national diagnostic reference levels for CTDI_vol_ for lumbar spine CT are reported to be up to 16 mGy [9,29]. In contrast, our proposed imaging protocol (RDCT-2) with the use of IRL6 results in a CTDI_vol_ of 2.8 ± 1.5 mGy (mean effective radiation dose: 1.2 mSv). One previous study addressing dose reduction in lumbar spine CT reported effective dose values even below 1 mSv (effective dose: 0.94 mSv; CTDI_vol_: 2.04 mGy; tube voltage: 120 kV) [30]. However, this study was performed in a single bovine phantom model in a water tank (AP: 22.0 cm; AL: 28.0 cm) that comprised only the lower thoracic and lumbar spine with all soft tissue (except the skin) preserved around the vertebrae. On average, the human cadavers in our study were bigger in size (AP: 24.5 ± 3.9 cm; AL: 37.7 ± 6.2 cm) and we chose a tube voltage of 140 kV, which previously has been recommended for the evaluation of osseous structures, hence resulting in a slightly higher CTDI_vol_ [31,32].

Our study has several limitations. Our first and major limitation is the lack of the intraindividual comparison of the actual dose exposure and diagnostic value of plain radiographs and our reduced-dose protocol. However, this direct comparison has already been performed by Alshamari, demonstrating the diagnostic superiority of CT. Furthermore, our study reported similar dose levels to plain radiographs, as previously described [1,18].

Secondly, even though this was not the aim of this study, we did not evaluate any bone pathologies. To account for this limitation, we used a subjective quality rating to evaluate clinically important and small anatomical structures. Nevertheless, future studies are required to investigate the reliability of RDCT when screening for bone pathologies.

Thirdly, we only investigated the effect of two levels of IR and FBP provided by one vendor. Thus, we potentially may not have selected the optimal strength of IR and our results might not be applicable for all scanner types and IR algorithms.

Fourth, the study was relatively small (20 cadavers) and skewed towards older subjects (mean 70 years) without known osseous pathologies. Thus, our observations are not necessarily applicable for an older population with significant bone loss nor a younger population. It is possible that a higher degree of bone loss is associated with an impaired image quality due to less high contrast structures within the bone. Regarding younger patients, we think it is generally easier to distinguish anatomic osseous structures in younger patients with higher bone density. This preliminary experimental study shows possibilities of high dose reduction in a specific context and can be used a reference point for further investigations of different osseous pathologies within different age groups.

Finally, all images were reconstructed at a slice thickness of 3 mm. Thinner slices, as used in other departments, increase image noise. Our choice to use reconstructions with a slice thickness of 3 mm was based on recommendations from the European guidelines on quality criteria for CT which state that a slice thickness of 2–5 mm is adequate for the evaluation of the osseous lumbar spine [33]. Also, a previous study about the effect of different slice thickness on detection of fracture in the cervical spine revealed no difference in detection of clinically important fractures [34].

## Conclusions

The current study demonstrates that highly reduced-dose lumbar spine CT is feasible using IR, resulting in an effective radiation dose of 1.2 mSv in this cadaver model.

Our proposed protocol has the potential to replace plain radiography of the lumbar spine in the future, since it offers high diagnostic value at levels of radiation exposure comparable to a two-view radiograph.
